# Genetic confounds of transgenerational epigenetic inheritance in mice

**DOI:** 10.1080/15592294.2024.2318519

**Published:** 2024-02-18

**Authors:** Daniel M. Sapozhnikov, Moshe Szyf

**Affiliations:** Department of Pharmacology and Therapeutics, Faculty of Medicine and Health Sciences, McGill University, Montreal, Quebec, Canada

**Keywords:** Epigenetics, transgenerational epigenetics, inheritance, genetics, mammals, transgenerational epigenetic inheritance, mouse

## Abstract

Transgenerational epigenetic inheritance in mammals remains a controversial phenomenon. A recent study by Takahashi et al. provides evidence for this mode of inheritance in mice by using a CRISPR/Cas9-based epigenetic editing technique to modify DNA methylation levels at specific promoters and then demonstrating the inheritance of the gain in methylation in offspring. In this technical commentary, we argue that the method used in the original study inherently amplifies the likelihood of genetic changes that thereafter lead to the heritability of epigenetic changes. We provide evidence that genetic changes from multiple sources do indeed occur in these experiments and explore several avenues by which these changes could be causal to the apparent inheritance of epigenetic changes. We conclude a genetic basis of inheritance cannot be ruled out and thus transgenerational epigenetic inheritance has not been adequately established by the original study.

## Main text

Transgenerational epigenetic inheritance refers to the transmission of epigenetic changes – typically, DNA methylation, histone modifications, and non-coding RNAs – across generations as a molecular mechanism for the potential impacts of a wide range of environmental exposures on health and disease susceptibility on unexposed descendants [1]. While transgenerational epigenetic inheritance has been thoroughly accepted as an observable phenomenon in plants and some animals, its existence in and importance in mammals continues to be debated [1]. In a recent major finding published in *Cell* [2], Takahashi et al. provide new evidence that at least artificially introduced epigenetic changes could be transmitted across generations in mice. In this study, Takahashi et al. disrupt unmethylated CpG islands in *Ankrd26* and *Ldlr* genes by the CRISPR/Cas9-mediated insertion of a large CpG-free cassette in mouse embryonic stem cells (mESCs), which leads to increased DNA methylation of the CpG islands. This hypermethylation persisted when the cassette was excised from the DNA and thereafter across several generations of mice derived from these modified mESCs. Though this elegant and well-executed study has been widely accepted as evidence of transgenerational epigenetic inheritance, this work expertly minimizes but does not incontrovertibly exclude the co-transmission of experimentally introduced genetic changes that could thus challenge the hypothesis of transgenerational epigenetic inheritance and reflect instead a simple genetic basis for inheritance. The genetic changes introduced by Takahashi et al. belong to at least three categories: direct mutations resulting from the recombination strategy, undefined CRISPR/Cas9 off-target mutagenesis, and the risk of clonal genetic heterogeneity. Here, focusing on the *Ankrd26* experiments by Takahashi et al., we present the rationale by which each can contribute to genetically heritable changes in *Ankdr26* expression and methylation and use publicly available data to provide evidence in support of the presence and consequences of each type of genetic change.

## Genetic changes accompany epigenetic changes

As excision-only PiggyBac transposase requires TTAA sequences at the end of two inverted terminal repeats (ITRs) in order to excise the DNA between them, Takahashi et al. elected to target the CpG-free cassette to a similar TTCT sequence near the transcription start site (TSS) and in the 5’ UTR of *Ankrd26*, such that, after excision of the cassette, targeted alleles (and later, modified offspring) could then be identified by a TTCT to TTAA mutation. This 2-bp region of mutation (CT to AA) is partially contained within the binding peaks of at least 217 potentially interacting transcription factors (Supplementary File 1) with available mouse ChIP-sequencing data across ENCODE and GEO databases (compiled with TFmapper) [3]. Therefore, the mutation can impact the binding of any number of transcription factors that could thus lead to DNA hypermethylation and gene repression and maintenance of this epigenetic state across generations. The fact that small nucleotide changes can affect gene expression and DNA methylation is a fundamental principle in genetics, defined as expression quantitative trait loci (eQTL) and methylation quantitative trait loci (mQTL). In fact, single nucleotide substitutions in the 5’ UTR of human *ANKRD26* are known to be functionally relevant; they modify *ANKRD26* expression and are the genetic cause of familial thrombocytopenia 2 (THC2) [4,5]. Takahashi et al. make an effort to address this possibility by producing a single ‘seamless’ (SL) clone, wherein a TTAA site (45-bp further in the 5’ UTR than the original TTCT site) was instead targeted, such that stable methylation can be introduced without a genetic change. However, the authors report that the clone carried the mutation on only one allele, while the other allele contained a CRISPR/Cas9-mediated deletion: thus, a genetic change was still introduced. Though the author’s report a nearly 50% hypermethylation of the CpG island in this SL clone, their approach for methylation detection does not discriminate alleles and it is therefore possible that only the allele with the deletion – and not the recombined allele – was hypermethylated. Moreover, even if the recombined allele was hypermethylated, the genetic change may work in *trans* by modifying *Ankrd26* transcriptional output, thereby potentially altering regulatory networks and activating compensatory pathways that similarly lead to *Ankrd26* hypermethylation/repression at both alleles.

An additional major genetic change introduced in this protocol was through the use of homology arms (required for CRISPR/Cas9-mediated recombination) that carried the C57BL/6J (B6) strain genotype. Since the experiments were performed in mESCs derived from B6/129 Sv hybrid mice, successful insertion of the CpG-free cassette also resulted in the replacement of 129 Sv allele with the B6 allele near the *Ankrd26* TSS, which Takahashi et al. acknowledge and detect by whole-genome sequencing (WGS). Inbred mouse strain has been repeatedly demonstrated to be the greatest driver of gene expression variance [6,7]. For reasons similar to those described above, these local nucleotide changes can behave as eQTL and/or mQTL and therefore the 129 Sv to B6 genotype switch may be sufficient to induce the observed hypermethylation, independently or in coordination with the insertion of the CpG-free cassette. Moreover, the change to B6 genotype at one of these positions (chr6:118,539,129 in mm39 genome) removes an alternative start codon that is present in the 129 Sv genotype and, though this 129 Sv protein product is out of frame with the Ankrd26 protein, its loss in edited cells is likely to alter translation of Ankrd26 from the mRNA by modifying the 5’ UTR and/or ribosomal binding and may lead to a regulatory feedback loop that modified *Ankrd26* expression and methylation.

**Figure 1.**
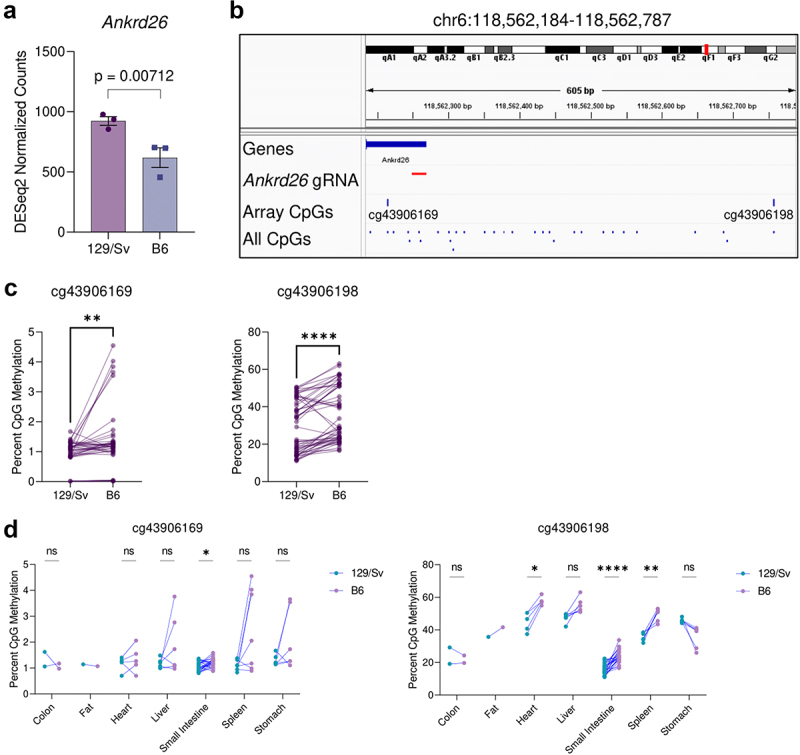
Strain-specific differences in Ankrd26 expression and methylation. (a) Comparison of the expression of Ankrd26 in 129Sv and B6 mEscs. Y-axis represents normalized RNA-seq gene-level counts quantified with the DESeq2 pipeline. The p-value was calculated by the DESeq2 pipeline, which uses the Wald test to compare expression between groups. (b) Schematic diagram of the Ankrd26 locus from the mm10 genome build which displays the Ankrd26 TSS and 5’UTR, the Ankrd26-targeted guide RNA (gRNA) used by Takahashi et al., two flanking CpGs which are present on the Infinium Mouse Methylation Array, and all CpGs in the region. (c) Methylation levels of cg43906169 (left) and cg43906198 (right) from the Infinium Mouse methylation Array in matched 129Sv-B6 pairs. (d) Same as (c) except matched pairs are separated by tissue. For (c) and (d) p-values were calculated by paired-tests (c) or multiple-paired t-tests with adjustment for multiple testing by the FDR method (D). ns indicates no statistically significant difference, *indicates *p* < 0.05, **indicates *p* < 0.01, and ****indicates *p* < 0.0001.

To determine if the B6 genotype is sufficient to explain hypermethylation and reduced expression, we used published mESC RNA-sequencing data which was specifically aimed at identifying strain-specific gene expression and eQTLs [6]. While this published dataset lists *Ankrd26* as a significant strain-specific gene across 8 strains, we re-analysed the data using only the two strains in question and found that *Ankrd26* expression was significantly reduced specifically in the B6 genetic background compared to the 129 Sv background when tested alone (unadjusted *p* = 0.007124816) and even under hypothesis-free genome-wide differential RNA-sequencing analysis (adjusted *p* = 0.019081) (Figure 1(a)). This suggests that a change from 129 Sv to B6 genotype may be sufficient to reduce expression of *Ankrd26*, and while it may be caused by more distant eQTLs, eQTLs that are close to the TSS – such as those introduced by the protocol – typically exhibit much larger effect sizes on expression [8].

We then used a mouse methylation array dataset comprising of over 1000 samples [9] to assess whether methylation of *Ankrd26* is also elevated in the B6 genome. Though there were no mESCs for both strains, data was available for numerous tissues and animal ages. To produce comparable datasets, we assigned 129 Sv and B6 pairs that were matched by tissue-of-origin and sex and, given multiple ages per matched tissue and sex, further matched by closest age, with a maximum age difference cut-off of 60 days, which yielded 54 matched pairs (108 samples, 39 female and 15 male pairs) across 7 tissues with a median age difference of 6 days and a maximum age difference of 35 days. Though there are no probes on the array that are directly in the CpG island studied by Takahashi et al., we assessed the methylation status of the two CpGs immediately flanking the CpG island and targeted site (Figure 1(b)). Here, we found that the B6 genome was indeed hypermethylated at both CpG sites, generally (Figure 1(c)), and this hypermethylation could be found at multiple tissues (Figure 1(d)).

In summary, the fact that genetic changes were invariably concomitant to epigenetic changes discounts hypermethylation as the only variable and casts doubt on the hypothesis of transgenerational epigenetic inheritance, allowing instead the possibility of a genetic basis for the observed inheritance.

## CRISPR/Cas9 off-target mutagenesis introduces additional genetic changes

The second source of potential genetic change is the fact that the initial insertion of the CpG-free cassette is dependent on CRISPR/Cas9, which has a high frequency of off-target mutagenesis [10]. This highlights a potential danger of numerous off-target mutations that occur in addition to the *Ankrd26* locus which may contribute to the observed epigenetic effects. Takahashi et al. provide evidence which supports no off-target mutagenesis of CRISPR/Cas9 in the form of WGS, from which they conclude that there are no single nucleotide variants (SNVs) or insertions/deletions (INDELs) within a 1 × 10^6^ bp range from the targeted site in *Ankrd26* nor in any predicted off-target targets based on a maximum of three mismatches. However, this narrow analysis in light of the availability of genome-wide data is concerning and represents an insufficiently thorough assessment of CRISPR/Cas9 off-target mutagenesis that is not typical for the field: while mutations within 1 × 10^6^ bp *Ankrd26* may indeed affect local hypermethylation and expression, there is no basis for discounting the possibility of the same effects resulting from off-target mutation elsewhere, including those that could occur in the genes encoding dozens of enzymes directly involved in modifying DNA methylation, a larger network of proteins that regulate the activity of these enzymes, and similarly, any transcription factors that regulate *Ankrd26* as well as their functional interactors, or across the vast regulatory non-coding genome that may regulate expression of *Ankrd26* or any of these proteins. The maximum of three mismatches is well below the threshold for off-target mutagenesis of CRISPR/Cas9; off-target cleavage of sites with as many as seven mismatches is widely reported, even in the absence of off-target cleavage of sites with fewer mismatches [11].

**Figure 2.**
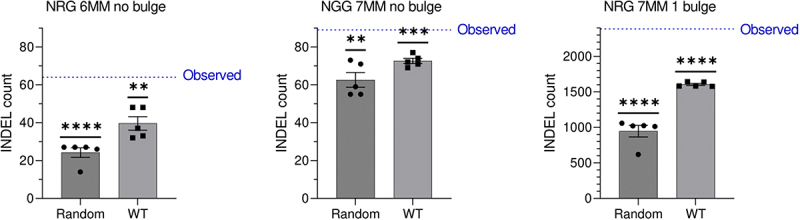
Indels in predicted CRISPR/Cas9 off-target regions in the edited HR1ex clone. All underlying data is provided in supplementary table 1. The blue line indicates the observed number of INDELs that are unique to HR1ex clone which occur in predicted off-target regions given the number of mismatches (MM), NGG or NRG PAM, and either no bulge or a 1-bp bulge between the gRNA and off-target site, indicated above each graph. Bars represent mean ± SEM of the number of unique HR1ex indels that occur in an equal number of equally sized random regions in the same clone with 5 independent randomizations (random condition) or the mean ± SEM of the number of WT INDELs (for which a number of INDELs equal to HR1ex unique INDELs were randomly selected 5 times from all WT INDELs) in the same predicted off-target regions (WT condition). Values were compared against the observed HR1ex INDEL count with a one-sample t-test. *indicates *p* < 0.05, **indicates *p* < 0.01, ***indicates *p* < 0.001, and **** indicates *p* < 0.0001.

While we confirm the absence of detectable variants when off-target mismatches are limited to three, expanding the off-target search space to NAG protospacer adjacent motifs (PAMs) with up to seven mismatches reveals a potential off-target landscape of CRISPR/Cas9. Though a raised number of quality-filtered INDELs unique to the edited (HR1ex) clone compared to that of INDELs unique to the wild-type (WT) clone (89,891vs 72,612) may not in itself be indicative of an off-target profile of CRISPR/Cas9 (note: unique INDELs are overestimated due to thresholding during INDEL calling), mapping INDELs to potential off-target sites identifies a large number of INDELs in regions with sequence similarity to the *Ankrd26* guide RNA (Supplementary Table S1). By comparing the number of HR1ex INDELs in potential off-target sites to HR1ex INDELs in an equal number of random regions of equal size, as wells as to WT INDELs in off-target regions, we conclude that at several categories of off-target sites, INDELs in random regions and WT INDELs are statistically significantly below the observed number of INDELs in HR1ex clones (Figure 2), suggesting a discernable off-target effect of CRISPR/Cas9. Furthermore, due to deficiencies in unique INDEL calling, we manually confirmed unique HR1ex INDELs in several of these regions with high similarity to the guide RNA sequence (Supplementary Figures S1–5) and confirmed absence of INDELs in these regions in the WT clone. It is important to note that off-target detection sensitivity with relatively low-coverage unreplicated WGS – the only data available from this study – might be underpowered to define with certainty the presence of off-target mutations caused by CRISPR/Cas9 that could otherwise be determined with deeper sequencing and more sensitive off-target detection methods.

Finally, though CRISPR/Cas9 editing can often lead to chromosome loss [12,13] and truncations of the arm on the edited chromosome [14–16] (in this case, chromosome 6), we found no evidence of either (Supplementary Figure 6A-B). Interestingly though, while examining chromosome 6 for 50-kb regions that exhibit altered read depths, we discovered three consecutive 50-kb regions with elevated read depth in the HR1ex clone (mm39: chr6:114450001–114500000, +31% average read depth in HR1ex; chr6:114500001–114550000, +19%; chr6:114550001–114600000, +23%) as compared to the WT clone. These same regions also had elevated coverage in the seamless (SL) clone (+43%, +38%, +35%) (Supplementary Figure 6C), suggesting either a reduced copy number in the WT clone or a consistent increased copy number when CRISPR/Cas9 was used in HR1ex and SL clones, as the region contained several predicted off-target sites (2 with 6 mismatches, NRG PAM; 3 with 7 mismatches, NGG PAM; 88 with 7 mismatches, NRG PAM). A similar example is evident at chr6:47600001–47650000 as well as numerous other similar differences across the different chromosomes (Supplementary Figure 7). Though we demonstrate this genetic difference in copy number between SL/HR1ex and WT clones, its biological source may not necessarily be CRISPR/Cas9 off-target mutagenesis. In the next section, we explore additional sources of genetic differences between edited and control clones.

## Clonal genetic heterogeneity is an additional source of genetic confounders

As cell lines are passaged in culture, individual cells accumulate genetic differences through spontaneous mutations and mitotic recombination as a function of passaging time. Moreover, in a phenomenon known as genetic drift, subclones which harbour variants that are adaptive under the selective pressures of cell culture can increase in frequency in the population. Therefore, clonal cell lines – including stem cell lines – derived from the same initial population have been repeatedly reported to show dramatic genetic [17–21] and, consequently, transcriptional and phenotypic differences [22,23]. Therefore, any observed effects that were attributed to transgenerational epigenetic inheritance – which relied on the comparison of edited (HR1ex) and control (WT) clonal lines – might instead be driven by genetic differences that emerge during prolonged culturing. While the single mESCs arising from an mESC culture could have already exhibited genetic differences at the time of the initial epigenetic editing treatment, the months of passaging in culture most probably resulted in accumulation of numerous genetic differences between the edited and control clones. The procedure described by Takahashi et al. involves two separate clonal expansion steps, first for isolation of clones in which the CpG-free cassette were integrated and again for isolation of clones where the cassette was excised. Again, genetic differences emerging through this long culturing protocol may drive hypermethylation and silencing of *Ankrd26* and its maintenance across generations independently of the predesigned epigenetic editing.

In fact, this risk may be specifically aggravated and directed by prolonged clonal expansion and passaging prior to excision – i.e., while the 4.3 kb CpG-free cassette disrupted the *Ankrd26* promoter and, likely, interfered with its expression – which may have directly led to the selection of subclones with genetic variants that compensated for the loss of *Ankrd26* expression and contributed to its stable hypermethylation and silencing; this is a common phenomenon observed when a gene is mutated and, combined with genetic drift, can lead to gene (expression) loss fixation in a population [24] and thus mimics but is certainly not epigenetic inheritance. In other words, the treatment might specifically direct heritable epigenetic silencing of the targeted gene but, almost paradoxically, not by the mechanism of transgenerational epigenetic inheritance. It is important to also consider here that the epigenetic editing technique used in this study is not in fact a bona fide epigenetic editing technique, but rather is a transient genetic editing technique.

**Figure 3.**
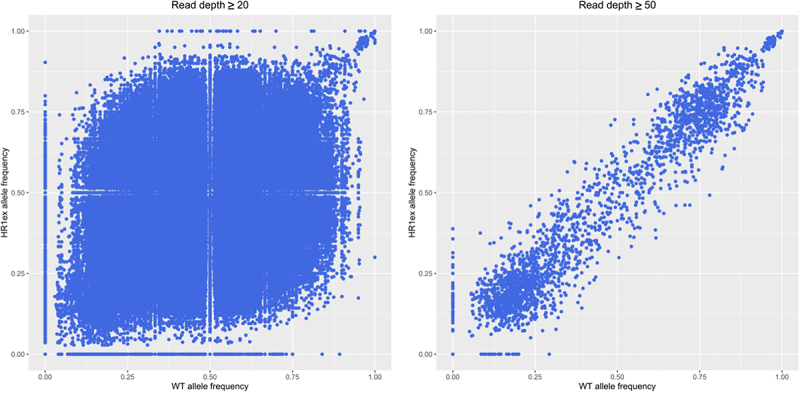
Altered allele frequencies in WT and HR1ex clones. Scatter plots of all alternate allele frequency at all positions with a minimal read depth of 20 (left) or 50 (right).

To provide evidence for this theory, we analysed the WGS data of the edited and control clones. In addition to nearly 100,000 INDELs unique to each clone, discussed above, there are 167,686 and 22,846 differentially genotyped biallelic SNVs, at read depths of 10 and 20 respectively, between the WT and edited HR1ex clonal cell lines. Genetic drift can be observed by a changed alternative allele frequency at these biallelic SNVs, which shows considerable variation at minimum read depths of 20 and 50 (Figure 3), including 2,687 SNVs with >50% change in allele frequency at a minimum read depth of 20. These extensive genetic differences in the HR1ex clone may potentially establish stable compensatory changes leading to *Ankrd26* loss fixation by modulating function or expression of genes related to *Ankrd26* such that *Ankrd26* expression is reduced and its promoter is hypermethylated. As a simplified example, 17 HR1ex-specific INDELs occur within the 5 known murine paralogs (from ENSEMBL) of *Ankrd26* and 1,875 HR1ex-specific INDELs occur within the 663 members of the Rho signalling pathway (Reactome ID: R-MMU-194315), which Ankrd26 is a member of.

We also identified larger scale chromosomal changes, both by analysing regions with altered read depth (as described above) and with the publicly available tool CNVnator [25]. CNVnator identified 1,706 deletions or duplications unique to the HR1ex clone (6,740 total HR1ex, 6,671 total WT). To identify particularly high-confidence sites, we compared CNVnator results with 50-kb bins that change in average read depth by more than 50%. Of 12 such 50-kb regions, 6 were also identified as unique HR1ex CNVs by CNVnator. This represents two genes: one deletion is in the *Ndst4* gene on chromosome 3, which contained 5 consecutive 50-kb deletions, each with a 53–55% reduced average sequencing depth, suggesting deletion on one allele. The remaining 50-kb region on chromosome 7 contains nearly the entire *Myo7a* gene and shows a similar 53% reduced sequencing depth. While these examples share no obvious connection to *Ankrd26*, they may result in global changes that affect *Ankrd26* expression and methylation. Additionally, copy number variants detected by CNVnator were found in 22 of the 663 members of the Rho pathway. We also identified 51 potential chromosomal translocations supported by >2 reads unique to the HR1ex clone. These genetic changes could contribute to maintenance of the hypermethylated state of *Ankrd26* across generations and therefore the transgenerational inheritance of this epigenetic state might have a genetic basis.

## Concluding remarks

The study by Takahashi et al. undoubtedly provides what is among the strongest evidence for transgenerational epigenetic inheritance in mammals to date, with an elegant experimental design that minimized the risk of genetic confounds to the highest levels allowed by the constraints of biology and current technology. Here, we argue that the persistent genetic confounds that cannot be circumvented preclude a high degree of certainty in the conclusion that transgenerational epigenetic inheritance was observed. Genetic differences between control and epi-edited clonal cell lines can in theory emerge from three potential sources: changes that are inherent to the protocol, CRISPR/Cas9 off-target mutagenesis, and clonal heterogeneity. We use publicly available data to provide evidence as to the existence and relevance of these factors in challenging the experimental design by Takahashi et al.

While clonal genetic heterogeneity would be difficult to disentangle from the protocol, the two types of genetic changes that are introduced as part of the recombination and CRISPR/Cas9 dependent technique could be addressed by replicating these experiments with an epigenetic editing protocol that does not introduce genetic changes, such as using a ‘dead’ Cas9 (dCas9) – which has no ability to cause mutation – fused to a methyltransferase domain for site-specific methylation of the *Ankrd26* locus, and investigating whether the ensuing hypermethylation can be transmitted transgenerationally. Unfortunately, while such an approach would resolve this issue, the lack of genetic changes could complicate the identification of offspring that acquired the epi-edited allele. Thus, the elegant approach by Takahashi et al. is well-suited to address transgenerational epigenetic inheritance, but simultaneously suffers from the inability to do so without introducing genetic confounds. In this regard, an important question is why DNA methylation deposited by dCas9-methyltransferase fusions is reported to be transient and is lost after expression of dCas9-methyltransferase is withdrawn [26], whereas the technique used by Takahashi et al. produces hypermethylation which is stable through cell divisions and developmental waves of epigenetic reprogramming; could it be because genetic changes accompany the epigenetic changes?

In the original study by Takahashi et al., the authors presented evidence of the phenomenon at a second locus. Here, we elected to focus on the *Ankrd26* example, but it stands to reason that a similar GTAC to TTAA mutation, the use of CRISPR/Cas9, and clonal selection could lead to similar genetic corollaries that confound the hypothesis of transgenerational epigenetic inheritance.

Finally, it is important to note that from a causality standpoint, numerous changes additional to hypermethylation co-occur: double-strand breaks and DNA repair machinery as well as histone changes induced by the disruption of this region by a 4.3 kb insertion may contribute to epigenetic silencing independently of DNA methylation changes and thus any inheritance of hypermethylation may be secondary to such changes. Takahashi et al. were not unaware of this, but it is important to restate that this approach does not measure the causality of the original DNA hypermethylation in transmitting the ensuing changes transgenerationally. Furthermore, the authors present lowly methylated clone HR7ex with a less dramatic downregulation (in their Figure S1G-H) as evidence that methylation is the cause of repressed expression, but this is purely correlative evidence.

The authors were aware of the risks of genetic confounds stemming from the TTAA to TTCC mutation and from CRISPR/Cas9 off-target mutagenesis and attempted to demonstrate that these risks were mitigated; yet the experiments were not sufficient in addressing these concerns, nor of the others discussed herein. In conclusion, while Takahashi et al. may have provided strong evidence of this phenomenon in mice, there remains in this study considerable risk of genetic confounds that should caution against accepting this as irrefutable proof of transgenerational epigenetic inheritance in mammals. Future studies face a difficult task in demonstrating bona fide transgenerational inheritance as the burden of proof inherently requires complete confidence in a lack of a genetic basis for heritability.

## Methods

### RNA-sequencing analysis

Raw gene-level RNA-seq counts from 129 Sv and B6 mESCs were downloaded from publicly available data (E-MTAB-7730). For B6 data, three technical replicates per each biological replicate were summed. Genome-wide differential expression analysis was performed with a default DESeq2 [27] (v. 1.38.3) pipeline in R after pre-filtering to keep only rows that have at least 10 total reads across all samples. For visualization of *Ankrd26* expression, normalized counts from the DESeq2 pipeline were exported to GraphPad Prism v9.4.1.

### Methylation analysis

129 Sv and B6 methylation data for the Infinium Mouse Methylation Array was downloaded from publicly available data (GSE184410). Sample metadata was used to define matched pairs on the basis of same tissue-of-origin and sex, and further by closest matching age, with a maximum age difference of 60 days. Statistical tests were performed with GraphPad Prism v9.4.1.

### SNV, INDEL, and structural variant analysis

Raw whole-genome sequencing data in FASTQ format for HR1ex and WT clones was downloaded from publicly available data from Takahashi et al. (GSE160847). Paired-end FASTQ files were trimmed for quality and adapter content using Trim Galore v0.6.7 with default parameters and then aligned to the mouse (mm39) reference genome using bowtie2 v2.4.5 [28] with default parameters. Resulting SAM files were converted to BAM format and unmapped reads were removed with samtools v1.16.1 [29]. BAM files were then sorted and duplicates were marked with Picard tools v2.18.29 and indexed with samtools. Read groups were added with GATK v4.2.6.1 [30]. SNVs and INDELs were called with GATK HaplotypeCaller using default parameters, followed by joint genotyping with GATK GenotypeGVCFs, and low-confidence calls were filtered using the following standard parameters: FS > 200.0, ReadPosRankSum < −20.0, QUAL < 30.0, QD < 2.0 for INDELs, and QD < 2.0, QUAL < 30.0, SOR > 3.0, FS > 60.0, MQ < 40.0, MQRankSum < −12.5, and ReadPosRankSum < −8.0 for SNVs. INDELs specific for each clone were identified by excluding overlapping INDEL positions in the other clone and requiring ≥10X coverage of the region in question in the other clone without evidence of any single read supporting an alternate allele. SNV allele frequency was calculated as the number of reads supporting the alternative allele divided by the read depth at the position, after all quality filtering. Chromosomal translocations with >2 reads supporting the translocation event were identified with breakdancer-max [31] using default parameters. CNVnator was run with default parameters.

### Off-target and read depth analyses

CRISPR/Cas9 off-targets were predicted using Cas-OFFinder v2.4 [32] and Cas-OFFinder-bulge v1.2 against the mm39 genome, using the parameters specified in the text. Read depth at each position was calculated by the bedtools v2.30.0 [33] genomecov function. 50-kb bins across the mm39 genome were created with the bedtools v2.30.0 make windows function and average coverage of each window was calculated by overlapping coverage data for each position with each bin using the bedtools v2.30.0 intersect function and then per-bin averages were calculated with the bedtools v2.30.0 merge function. Using the bedtools v2.30.0 getfasta function, bins with > 100 undefined bases (Ns) were discarded. All coverage plots were plotted with the ggplot2 package in R.

## Supplementary Material

Supplementary_File_1.xlsx

-)Supplementary_Info.docx

## Data Availability

There was no original data generated in this study.
