# Supplementary material for: Genetic confounds of transgenerational epigenetic inheritance in mice
Source: Epigenetics. 2024 Feb 18;19(1):2318519. doi: 10.1080/15592294.2024.2318519 (PMC10878023; doi:10.1080/15592294.2024.2318519)
Supplement: -)Supplementary_Info.docx [file KEPI_A_2318519_SM6835.docx]

|  |  |  |  |  | Unique HR1ex INDELs in | | | | | All WT INDELs in Off- | | | |  |
| --- | --- | --- | --- | --- | --- | --- | --- | --- | --- | --- | --- | --- | --- | --- |
|  |  |  |  |  | random regions | | |  |  | Target regions | | |  |  |
|  |  |  |  | Unique |  |  |  |  |  |  |  |  |  |  |
|  |  |  | Total | HR1ex |  |  |  |  |  |  |  |  |  |  |
|  |  |  | Off- | INDELs |  |  |  |  |  |  |  |  |  |  |
|  |  |  | Target | in Off- |  |  |  |  |  |  |  |  |  |  |
| MM | PAM | Bulge | Regions | Target | 1 | 2 | 3 | 4 | 5 | 1 | 2 | 3 | 4 | 5 |
| **3** | **NGG** | **1** | **66** | **0** | **0** | **1** | **0** | **1** | **0** | **0** | **0** | **0** | **0** | **0** |
| **3** | **NGG** | **0** | **8** | **0** | **0** | **0** | **0** | **0** | **0** | **0** | **0** | **0** | **0** | **0** |
| **3** | **NRG** | **1** | **137** | **0** | **1** | **0** | **1** | **0** | **0** | **0** | **0** | **0** | **1** | **0** |
| **3** | **NRG** | **0** | **15** | **0** | **0** | **0** | **0** | **0** | **0** | **0** | **0** | **0** | **0** | **0** |
| 4 | NGG | 1 | 919 | 1 | 0 | 0 | 1 | 0 | 1 | 0 | 1 | 1 | 0 | 0 |
| 4 | NGG | 0 | 97 | 0 | 0 | 1 | 0 | 1 | 0 | 0 | 0 | 0 | 0 | 0 |
| 4 | NRG | 1 | 2257 | 3 | 4 | 3 | 4 | 3 | 2 | 1 | 1 | 1 | 1 | 1 |
| 4 | NRG | 0 | 205 | 0 | 1 | 0 | 0 | 0 | 0 | 0 | 0 | 0 | 0 | 0 |
| **5** | **NGG** | **1** | **9725** | **8** | **7** | **20** | **7** | **20** | **4** | **12** | **7** | **12** | **7** | **7** |
| **5** | **NGG** | **0** | **966** | **2** | **1** | **0** | **0** | **0** | **0** | **3** | **4** | **2** | **0** | **2** |
| **5** | **NRG** | **1** | **24782** | **29** | **33** | **32** | **23** | **31** | **17** | **24** | **20** | **30** | **28** | **20** |
| **5** | **NRG** | **0** | **2821** | **10** | **4** | **2** | **3** | **2** | **0** | **2** | **3** | **4** | **6** | **4** |
| 6 | NGG | 1 | 72658 | 83 | 74 | 63 | 91 | 63 | 49 | 61 | 59 | 74 | 81 | 64 |
| 6 | NGG | 0 | 8336 | 17 | 8 | 7 | 7 | 7 | 4 | 9 | 4 | 10 | 8 | 11 |
| 6 | NRG | 1 | 185362 | 229 | 237 | 174 | 201 | 175 | 115 | 185 | 164 | 215 | 228 | 198 |
| 6 | NRG | 0 | 21947 | 64 | 27 | 27 | 26 | 27 | 14 | 32 | 33 | 48 | 37 | 48 |
| **7** | **NGG** | **1** | **425097** | **722** | **445** | **478** | **474** | **448** | **268** | **518** | **536** | **537** | **556** | **559** |
| **7** | **NGG** | **0** | **53463** | **89** | **59** | **55** | **73** | **55** | **51** | **77** | **74** | **72** | **69** | **71** |
| **7** | **NRG** | **1** | **966696** | **2386** | **1012** | **1059** | **1021** | **1024** | **618** | **1576** | **1583** | **1638** | **1642** | **1582** |
| **7** | **NRG** | **0** | **139658** | **552** | **141** | **158** | **150** | **148** | **98** | **342** | **363** | **330** | **337** | **334** |

**Supplementary Table 1**. INDELs in CRISPR/Cas9 off-target regions predicted by Cas-OFFinder.

1. indicates the maximum number of mismatches between the *Ankrd26* gRNA and the off-target site: increasing mismatch numbers are inclusive of all regions with fewer mismatches. PAM indicates the protospacer adjacent motif for CRISPR/Cas9, which could be NGG or NAG (R indicates A or G; NRG includes NAG and NGG). The “Bulge” column indicates whether or not a 1-bp bulge between gRNA and off-target DNA was allowed (1) or not (0). Unique HR1ex INDELs were also quantified in an equal number of equally-sized regions per each off-target region list, randomly selected 5 times. A subset of WT INDELs (not necessarily unique to wild-type) equal in number to the unique HR1ex INDEL number were also randomly selected 5 times and quantified in the off-target regions for each condition.


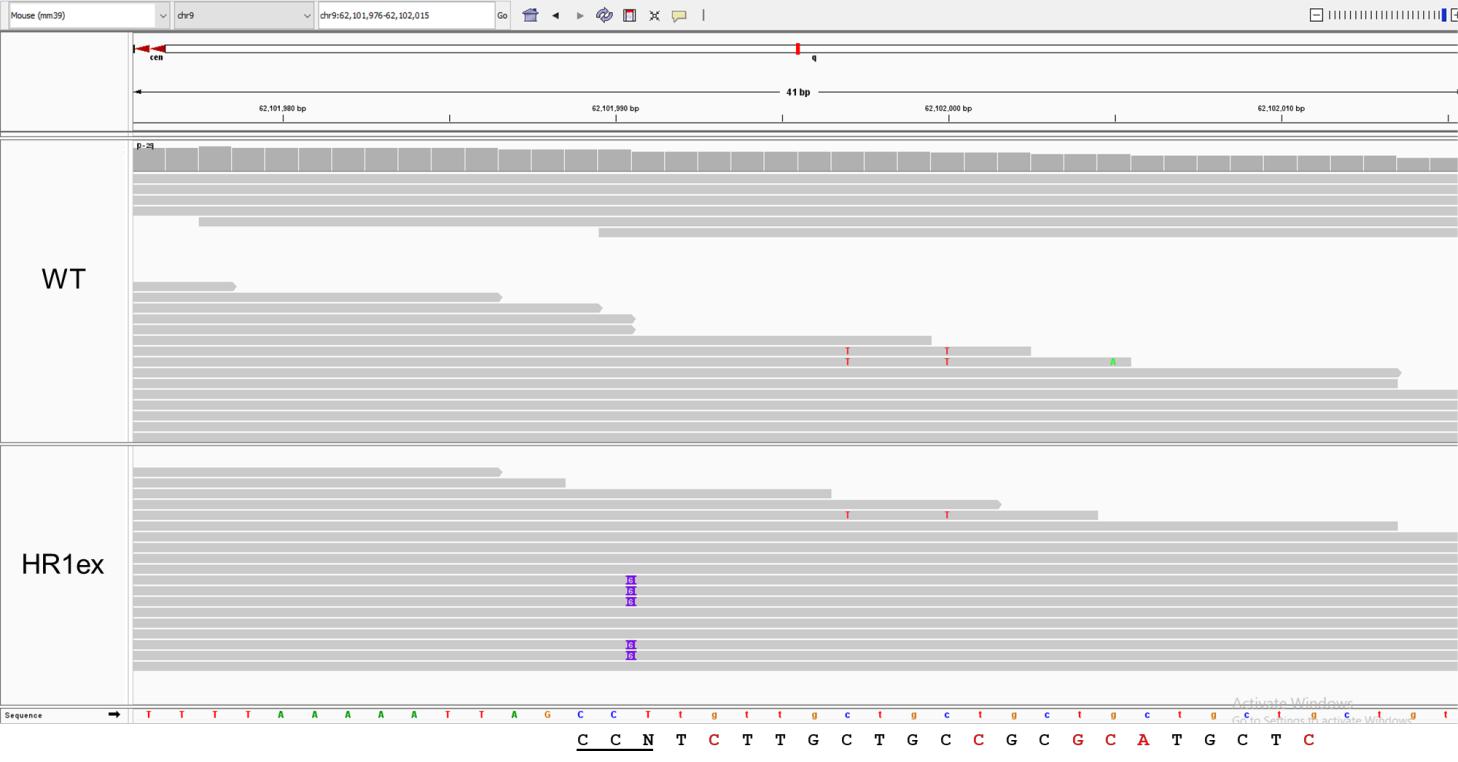


**Supplementary Figure 1**. Genome browser view of a predicted off-target CRISPR/Cas9 region that shows INDELs unique to the HR1ex clone and absent in the WT clone. Aligned reads are displayed with an apparent 6-bp insertion in the HR1ex clone. The reverse complement of the *Ankrd26*-targeting gRNA is shown below. Mismatches between the gRNA and the off-target site are depicted in red. CCN represents the reverse complement of the NGG PAM and is underlined.


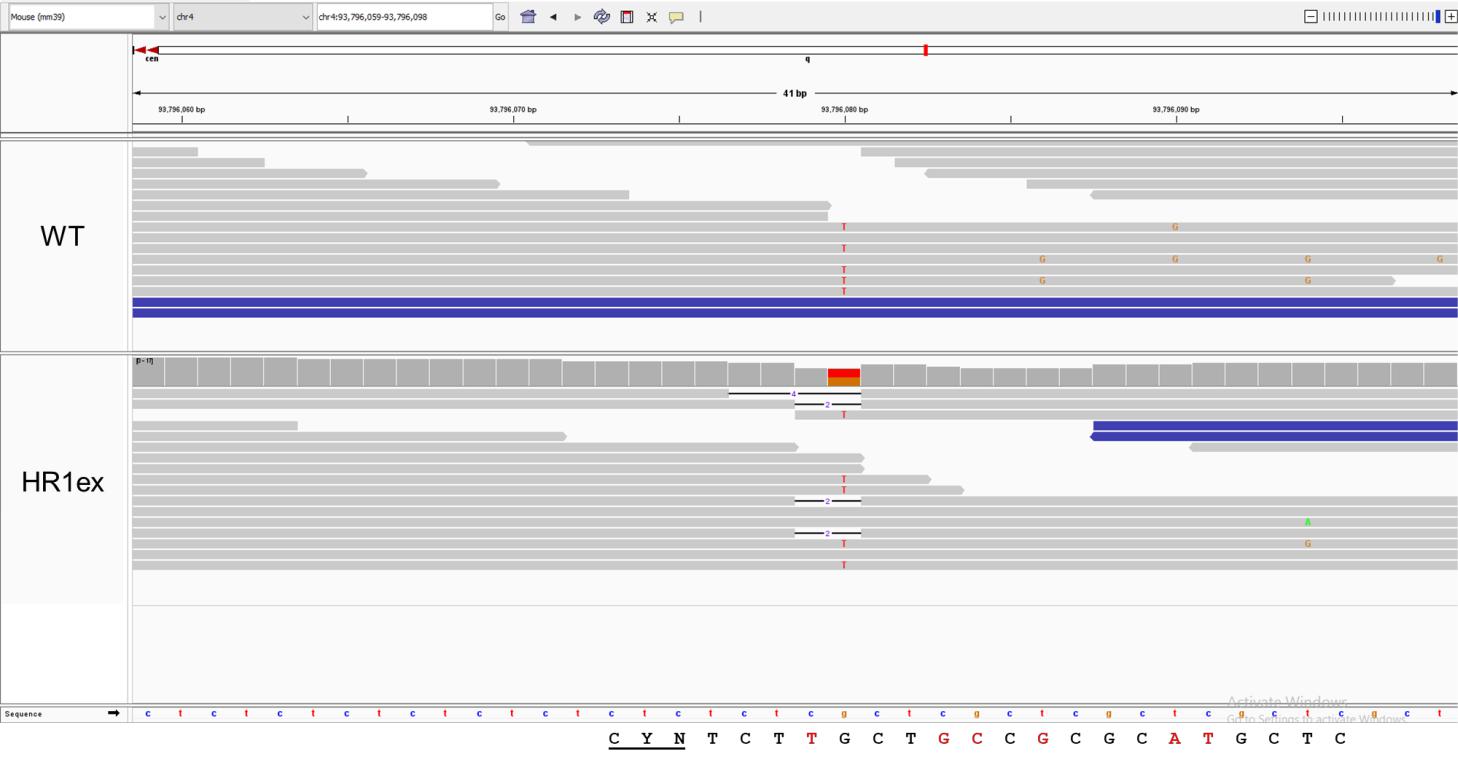


**Supplementary Figure 2**. Genome browser view of a predicted off-target CRISPR/Cas9 region that shows INDELs unique to the HR1ex clone and absent in the WT clone. Aligned reads are displayed with apparent 2-bp and 4-bp deletions in the HR1ex clone. The reverse complement of the *Ankrd26*-targeting gRNA is shown below. Mismatches between the gRNA and the off-target site are depicted in red. CYN represents the reverse complement of the NRG PAM and is underlined.


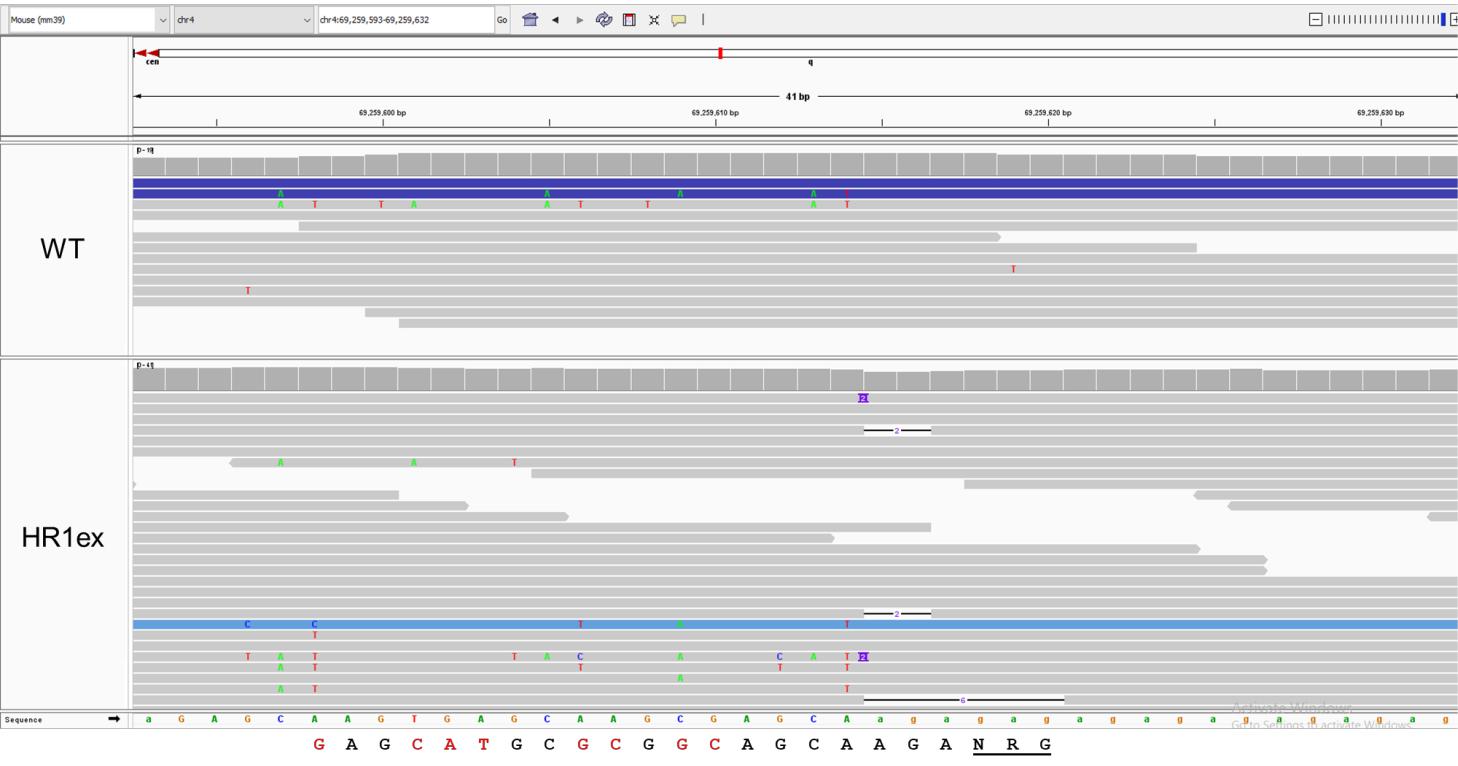


**Supplementary Figure 3**. Genome browser view of a predicted off-target CRISPR/Cas9 region that shows INDELs unique to the HR1ex clone and absent in the WT clone. Aligned reads are displayed with apparent 2-bp and 6-bp deletions in the HR1ex clone. The sequence of the *Ankrd26*-targeting gRNA is shown below. Mismatches between the gRNA and the off-target site are depicted in red. NRG is underlined and represents the PAM.


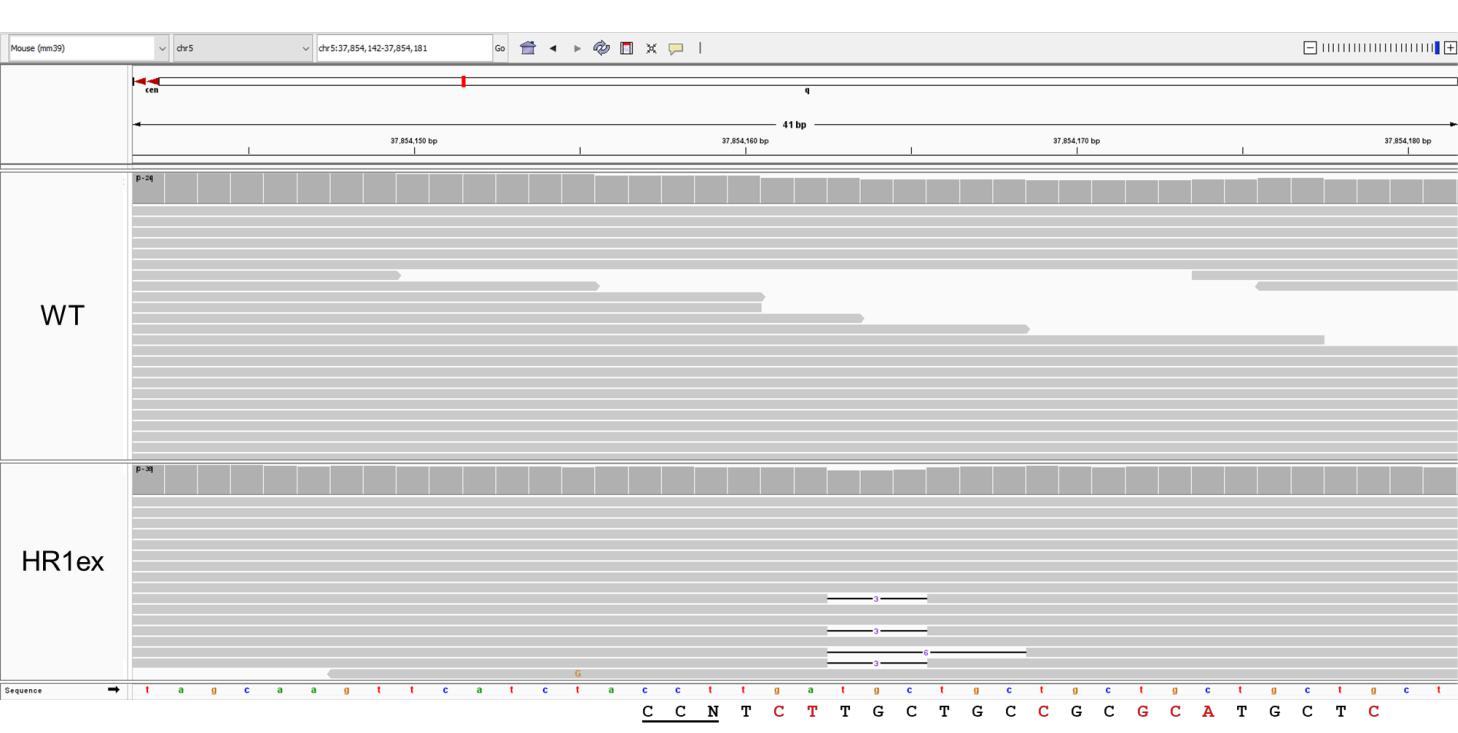


**Supplementary Figure 4**. Genome browser view of a predicted off-target CRISPR/Cas9 region that shows INDELs unique to the HR1ex clone and absent in the WT clone. Aligned reads are displayed with apparent 3-bp and 6-bp deletions in the HR1ex clone. The reverse complement of the *Ankrd26*-targeting gRNA is shown below. Mismatches between the gRNA and the off-target site are depicted in red. CCN represents the reverse complement of the NGG PAM and is underlined.


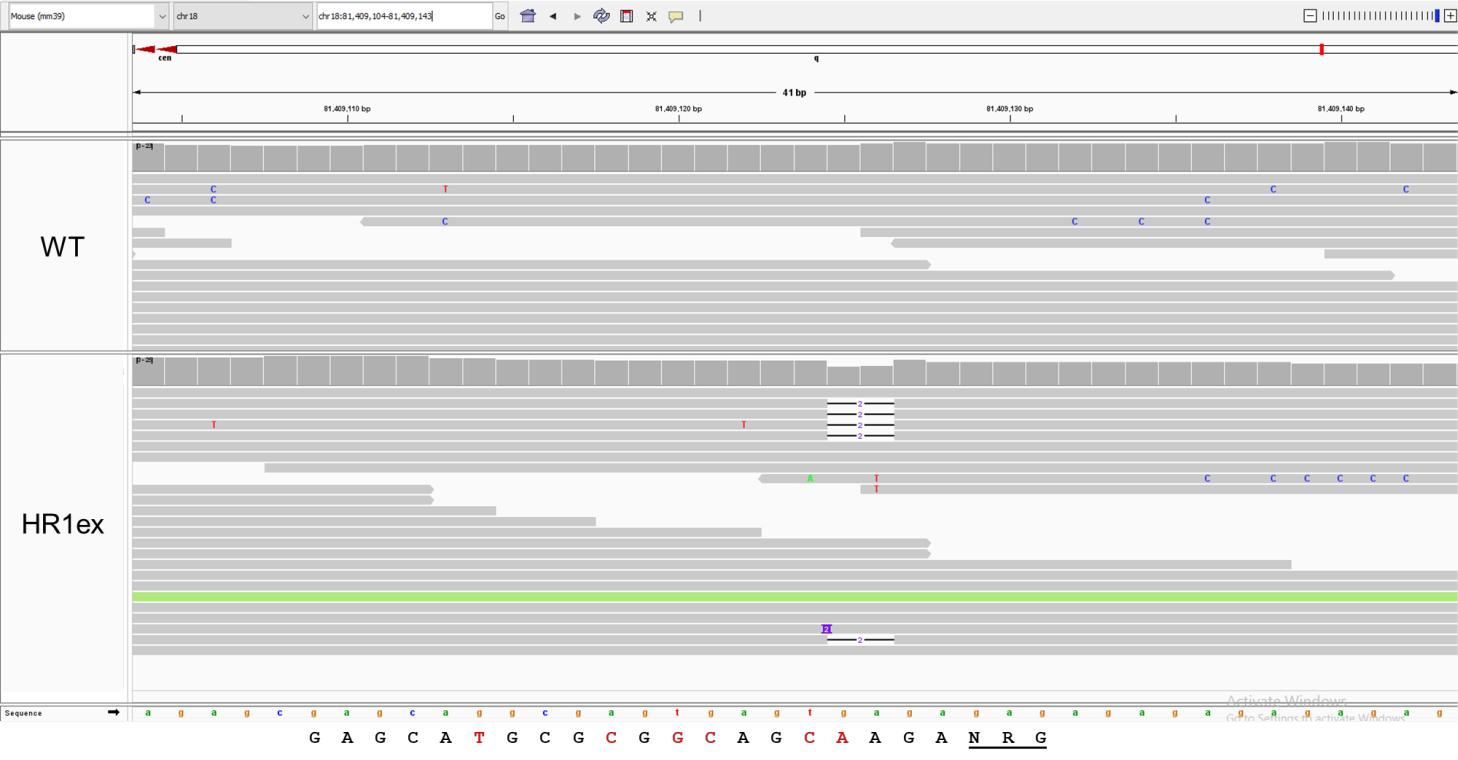


**Supplementary Figure 5**. Genome browser view of a predicted off-target CRISPR/Cas9 region that shows INDELs unique to the HR1ex clone and absent in the WT clone. Aligned reads are displayed with an apparent 2-bp deletion in the HR1ex clone. The sequence of the *Ankrd26*-targeting gRNA is shown below. Mismatches between the gRNA and the off-target site are depicted in red. NRG is underlined and represents the PAM.

A


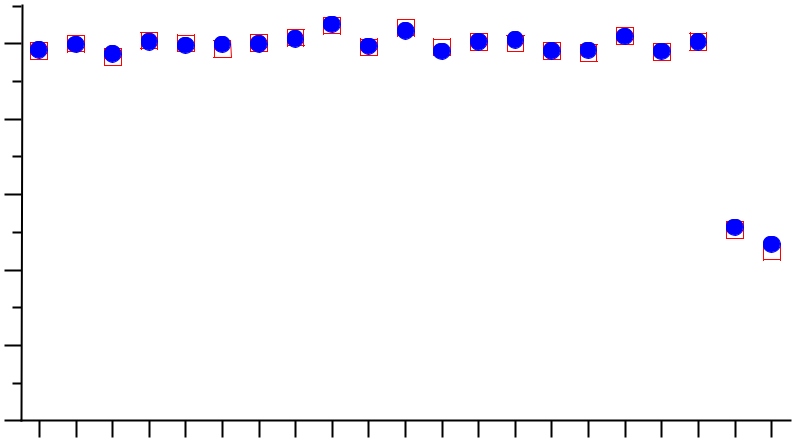


| PercentAverageAutosomalCoverage | 100 |  |
| --- | --- | --- |
|  | 80 |  |
|  |  |  |
|  | 60 |  |
|  | 40 |  |
|  | 20 |  |
|  | 0 |  |

|  |  | 2 3 4 5 6 | | | 7 | 8 | 9 | 0 | 1 2 3 | 4 | 5 | 6 | |  | 7 8 | 9 |  | Y |
| --- | --- | --- | --- | --- | --- | --- | --- | --- | --- | --- | --- | --- | --- | --- | --- | --- | --- | --- |
|  | r1 r | |  | r r r | r | r | r | 1 1 1 1 | | 1 | 1 1 | |  | 1 1 | |  | rX r | |
| h | |  | hr h h h h h h r r r r r r r | | | | | | | | |  | r | |  |  | h |  |
| c |  | ch c c | | c c c | c c | | h | h | h h h h h | | | h | |  | hr hr1 ch c | | |  |
|  |  |  |  |  |  |  | c | c | c c c | c c c | | |  | c c | |  |  |  |

WT


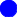


HR1ex


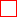


B


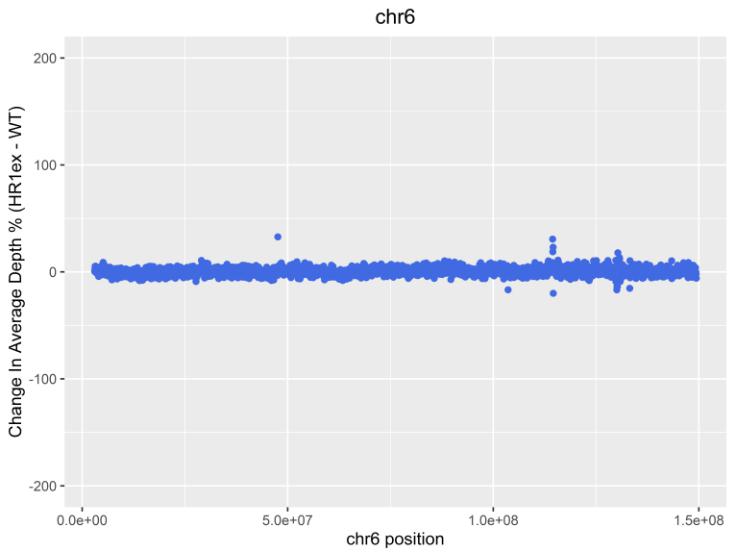


*

C

| SL |
| --- |

*


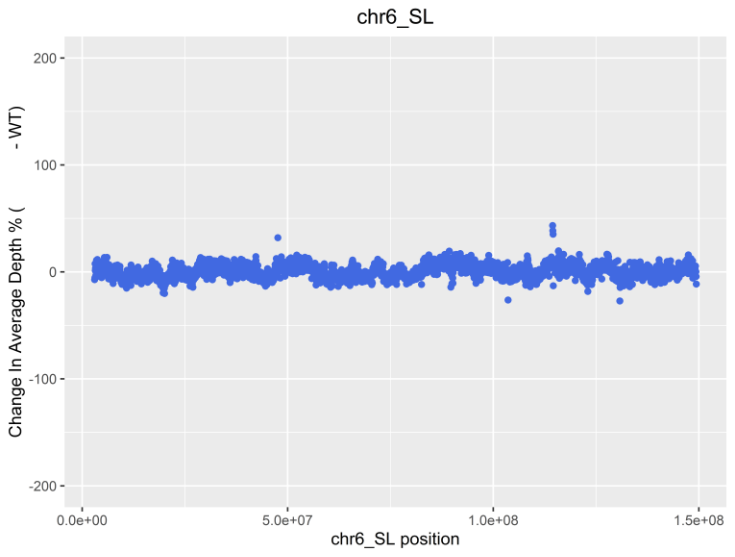


**Supplementary Figure 6**. Chromosomal differences between edited and wild-type clones. (A) Percent average read depth per chromosome, calculated as the average read depth of each position per chromosome divided by the average read depth for all positions in all chromosomes excluding chrX and chrY. No obvious differences are apparent, suggest no chromosomal loss. (B) Average read depth as in (A) but separated into 50-kb bins across chromosome 6 and divided by average read depth at all positions of chromosome 6 only and shown as change between HR1ex and WT clones. Regions with any stretch of Ns greater than 100-bp were excluded. The asterisks mark the region discussed in the text. (C) As in (B) but for the SL clone.


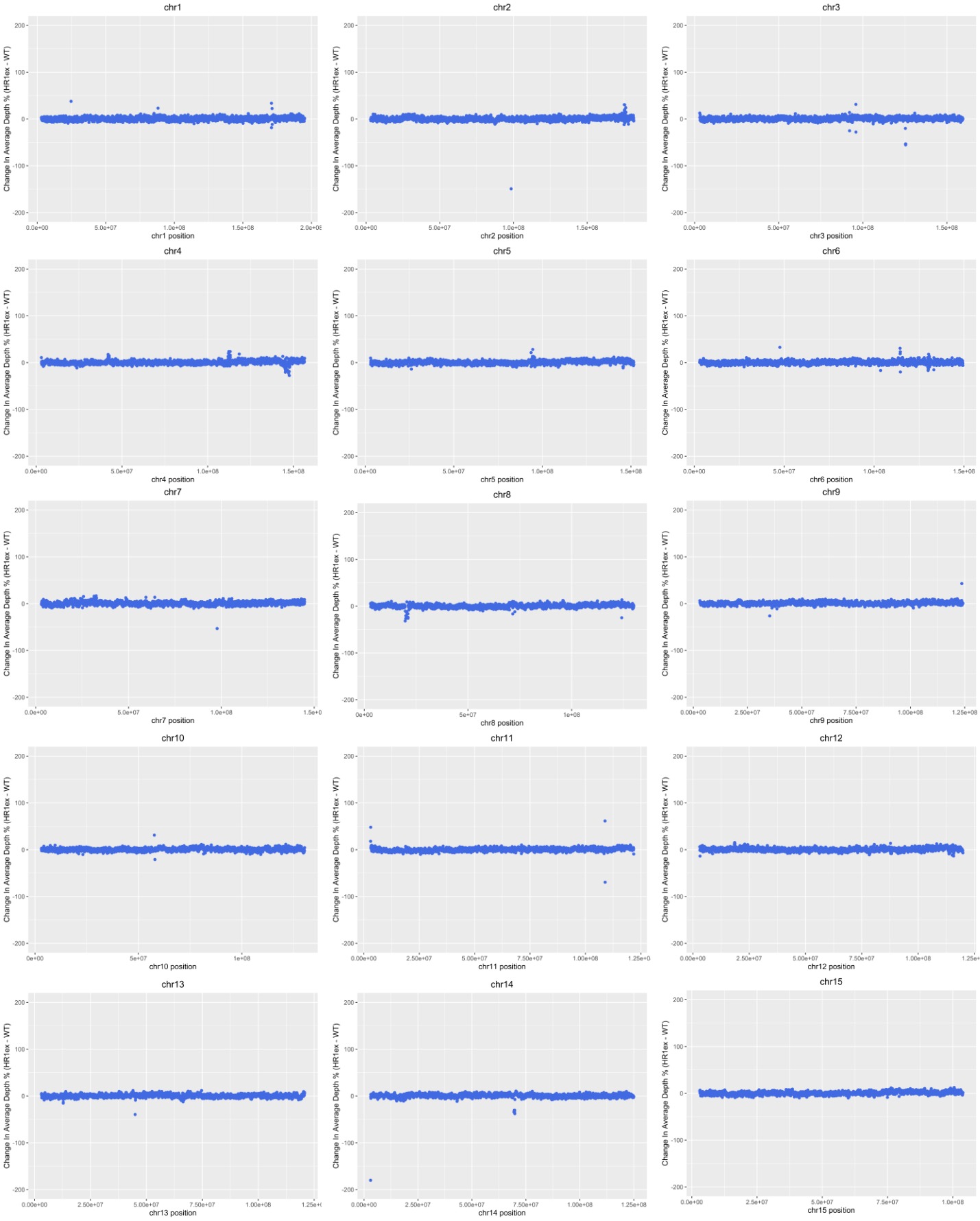


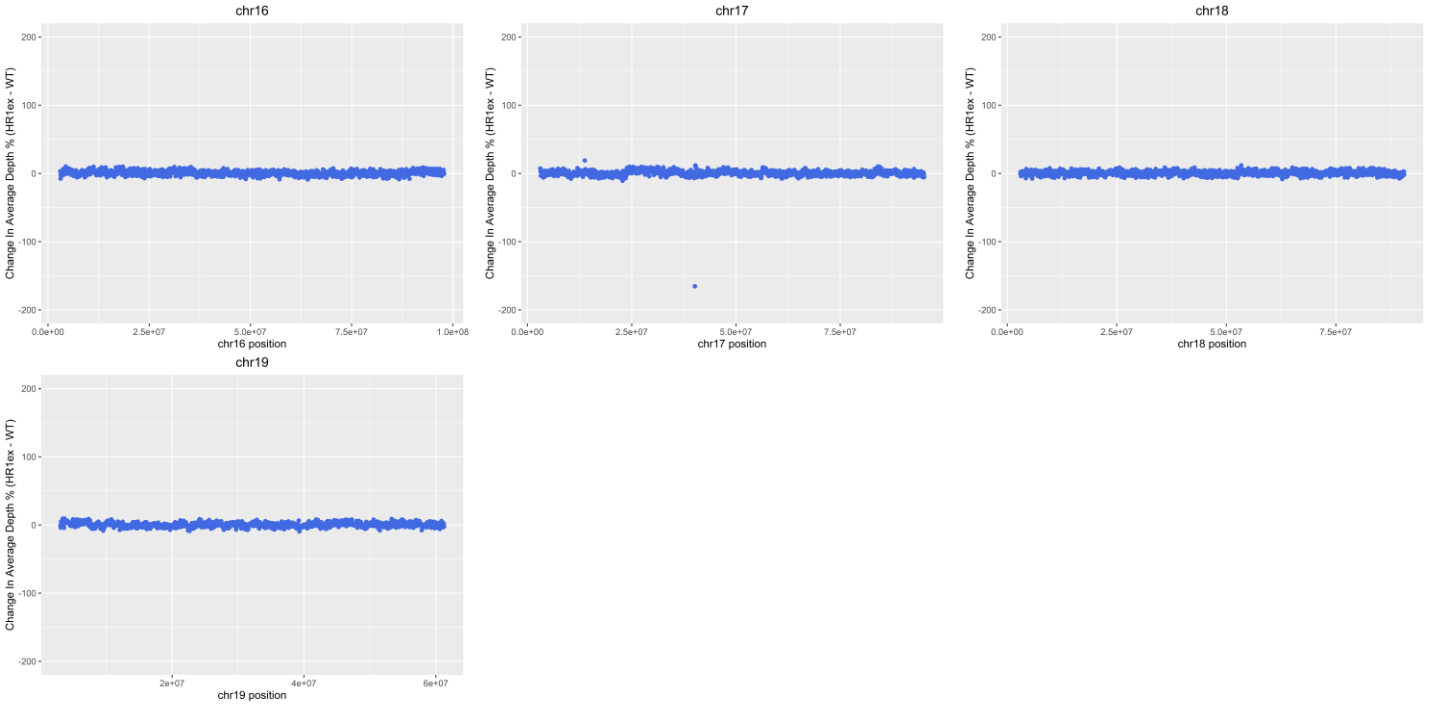


**Supplementary Figure 7**. As in Supplementary Figure 6B-C, average read depth over 50-kb bins across all autosomes and divided by average read depth at all positions of each chromosome and shown as change between HR1ex and WT clones. Regions with any stretch of Ns greater than 100-bp were excluded.
